# α-Solanine Inhibits Invasion of Human Prostate Cancer Cell by Suppressing Epithelial-Mesenchymal Transition and MMPs Expression

**DOI:** 10.3390/molecules190811896

**Published:** 2014-08-11

**Authors:** Kun-Hung Shen, Alex Chien-Hwa Liao, Jui-Hsiang Hung, Wei-Jiunn Lee, Kai-Chieh Hu, Pin-Tsen Lin, Ruei-Fang Liao, Pin-Shern Chen

**Affiliations:** 1 Division of Urology, Department of Surgery, Chi Mei Medical Center, Tainan 710, Taiwan; E-Mails: robert.shen@msa.hinet.net (K.-H.S.); ch159485@ms7.hinet.net (A.C.-H.L.); 2 Department of Optometry, College of Medicine and Life Science, Chung Hwa University of Medical Technology, Tainan 717, Taiwan; 3 Department of Urology, Taipei Medical University, Taipei 110, Taiwan; 4 Department of Senior Citizen Service Management, Chia Nan University of Pharmacy & Science, Tainan 717, Taiwan; 5 Department of Biotechnology, Chia Nan University of Pharmacy & Science, Tainan 717, Taiwan; E-Mails: hung86@mail.chna.edu.tw (J.-H.H.); apple11763@yahoo.com.tw (K.-C.H.); tina2572001@gmail.com (P.-T.L.); zeroadolz@gmail.com (R.-F.L.); 6 Department of Urology, Wan Fang Hospital, Taipei Medical University, Taipei, Taiwan; E-Mail: lwj5905@gmail.com

**Keywords:** α-solanine, invasion, EMT, matrix metalloproteinase, microRNA

## Abstract

α-Solanine, a naturally occurring steroidal glycoalkaloid found in nightshade (*Solanum nigrum* Linn.), was found to inhibit proliferation and induce apoptosis of tumor cells. However, the mechanism involved in suppression of cancer cell metastasis by α-solanine remains unclear. This study investigates the suppression mechanism of α-solanine on motility of the human prostate cancer cell PC-3. Results show that α-solanine reduces the viability of PC-3 cells. When treated with non-toxic doses of α-solanine, cell invasion is markedly suppressed by α-solanine. α-Solanine also significantly elevates epithelial marker E-cadherin expression, while it concomitantly decreases mesenchymal marker vimentin expression, suggesting it suppresses epithelial-mesenchymal transition (EMT). α-Solanine reduces the mRNA level of matrix metalloproteinase-2 (MMP-2), MMP-9 and extracellular inducer of matrix metalloproteinase (EMMPRIN), but increases the expression of reversion-inducing cysteine-rich protein with kazal motifs (RECK), and tissue inhibitor of metalloproteinase-1 (TIMP-1) and TIMP-2. Immunoblotting assays indicate α-solanine is effective in suppressing the phosphorylation of phosphatidylinositide-3 kinase (PI3K), Akt and ERK. Moreover, α-solanine downregulates oncogenic microRNA-21 (miR-21) and upregulates tumor suppressor miR-138 expression. Taken together, the results suggest that inhibition of PC-3 cell invasion by α-solanine may be, at least in part, through blocking EMT and MMPs expression. α-Solanine also reduces ERK and PI3K/Akt signaling pathways and regulates expression of miR-21 and miR-138. These findings suggest an attractive therapeutic potential of α-solanine for suppressing invasion of prostate cancer cell.

## 1. Introduction

Nightshade (*Solanum nigrum* Linn.) has been used as a herbal plant in Southeast Asia. Its extract can induce growth inhibition and apoptosis in breast cancer and hepatoma cells [1,2,3]. In addition, a water extract of the plant suppresses melanoma metastasis [4]. α-Solanine, a trisaccharide glycoalkaloid, is one of the main steroidal glycoalkaloids in Solanaceae family species such as nightshade and potato (*Solanum tuberosum* L.) [2,5]. Recent studies have demonstrated that α-solanine inhibits the growth of human colon, liver, cervical, lymphoma, and stomach cancer cells [6,7,8]. α-Solanine also exerts chemoprotective and chemotherapeutic effects in an animal model of breast cancer through induction of apoptosis, and inhibition of cell proliferation and angiogenesis [9]. In addition, α-solanine hinders migration and invasion of human melanoma cells [10]. Therefore, α-solanine may possess the potential for cancer chemotherapeutic action.

Prostate cancer is one of the most commonly diagnosed tumors in men and is the second leading cause of cancer mortality in the United States [11]. Although early stage prostate cancer can be treated with surgery and androgen-deprivation therapy, there is no effective therapy for the treatment of metastatic and malignant hormone refractory prostate cancer (HRPC) [12,13]. Thus, developing novel approaches for treatment of prostate cancer is necessary. In view of the high morbidity and mortality rates caused by advanced prostate cancer cell with highly invasive potential, inhibition of invasion and metastasis may be a good approach to treatment of HRPC.

Cancer metastasis is a highly coordinated and sequential process. Cells initially detach from the primary tumor and degrade the local extracellular matrix (ECM), followed by penetrating through the basement membrane and into capillary or lymphatic vessels, then invasion into new tissue and finally growth to form distant tumor [14,15]. Epithelial-mesenchymal transition (EMT) plays an important role during tumor dissemination by endowing cancer cells with greater motility and invasiveness [16,17]. It is typically characterized by decrease in expression of epithelial markers such as E-cadherin, and increase in expression of mesenchymal markers such as vimentin [18]. Moreover, the expression and secretion of several ECM-degrading proteolytic proteases play an important role in promoting the process of metastasis. Matrix metalloproteinases (MMPs), a family of Zn-dependent endopeptidases, are the major proteases that participate in tumor cell migration, spreading, tissue invasion and metastasis [19]. Of these MMPs, MMP-2 and MMP-9 are key enzymes and contribute to the process of metastasis [20,21]. The activation of these enzymes is associated with increased tumor metastasis, which suggests a central functional role for these proteases in the metastatic process [22]. 

In addition, ECM degradation during tumor metastasis is controlled by other proteins, such as extracellular inducer of matrix metalloproteinase (EMMPRIN), reversion-inducing, cysteine-rich protein with Kazal motif (RECK) and tissue inhibitor of metalloproteinases (TIMPs). EMMPRIN contributes to modify the tumor microenvironment by stimulating proteinases and angiogenic factors in tumor and stromal cells. EMMPRIN also plays a crucial role in the invasion and metastasis processes of prostate cancer cells by activating MMPs [23]. RECK acts as a negative regulator of tumor invasion and metastasis by suppressing MMP-2 and MMP-9 activities [24]. The lower expression of RECK is frequently correlate with higher invasiveness and poor prognosis [25]. The activities of most MMPs are also regulated by their endogenous tissue inhibitors TIMPs. The proteolytic activity of tumor cells is determined by the balance between MMP and TIMP levels [26]. 

MicroRNAs (miRNAs) are short noncoding RNAs that control the expression of target genes by repressing mRNA stability and translation. miRNAs are involved in several important biological functions such as cellular proliferation, differentiation as well as tumor initiation and progression [27,28]. Some miRNAs act as oncomirs that function as oncogenes or tumor suppressors [29,30]. For example, miR-21 serves as an oncomir and is up-regulated in many tumors including breast, colon, prostate and pancreas [31]. miR-21 regulates cancer cell proliferation, migration, invasion and metastasis by targeting different molecules such as Pdcd4 [32], PTEN [33] and RECK [34]. Recent study also indicated that antagonism of miR-21 reversed EMT, suggesting the role of miR-21 on regulating EMT [35]. miR-138 has been thought to function as a tumor suppressor. Deregulation of miR-138 is observed in a variety of cancer types, including thyroid carcinoma, head and neck squamous cell carcinoma and ovarian cancer [36,37,38]. miR-138 suppresses cell migration and invasion by targeting RhoC, ROCK2 [4] as well as EMT-related genes such as vimentin, ZEB2 and EZH2 [39]. 

Mitogen-activated protein kinase (MAPK) pathway is known to participate in various signaling cascades that play an important regulatory role in cell growth, apoptosis, differentiation, and metastasis [40]. Extracellular signal regulating kinase (ERK1/2) and c-Jun N-terminal kinase (JNK), two major mammalian MAP kinases, have been implicated in cell migration and proteinase induction [41]. Expression of MMPs is primarily regulated by ERK1/2 and JNK signaling pathways [19]. The inhibition of the MAPK pathway may potentially prevent angiogenesis, proliferation, invasion and metastasis in a wide range of tumors [42,43,44]. In addition, phosphatidylinositide-3 kinase (PI3K) and Akt signaling pathway are involved in the regulation of metastasis, cell adhesion and cell survival [45,46]. The inhibition of MAPK and PI3K/Akt pathways may potentially prevent cancer cell proliferation, invasion and metastasis [43,44]. Moreover, inhibitors of PI3K/Akt and ERK have been shown to reverse EMT [35]. Thus, PI3K/Akt and MAPK pathways are potential targets for therapeutic strategies of tumors. 

Although α-solanine exerts anti-carcinogenic potential against various cancer cell lines, the mechanism involved in inhibition of tumor metastasis by α-solanine remains unclear. This work determined the inhibitory effect and the molecular mechanisms of α-solanine on cell invasion *in vitro*. Since human prostate cancer PC-3 cells possess highly invasive and metastatic activity [47], they are suitable for studying the molecular mechanisms involved in cancer metastasis, and evaluating the responses for chemotherapeutic agents. Cell invasion is a coordinated process including EMT, increase of cell motility, and proteolysis of surrounding ECM. Thus we investigate the effect of α-solanine on EMT, cell migration, invasion and MMP-2/9 expression. In addition, EMT, cell migration/invasion and MMP-2/9 expression are controlled by PI3K/Akt and MAPK pathways as well as miR-21 and miR-138. We explore the effect of α-solanine on PI3K/Akt, MAPK pathways and expression of miR-21 and miR-138. This study obtains evidence that α-solanine can suppress the invasion of PC-3 cells.

## 2. Results and Discussion

### 2.1. Cytotoxic Effect of α-Solanine in PC-3 Cells

**Figure 1:**
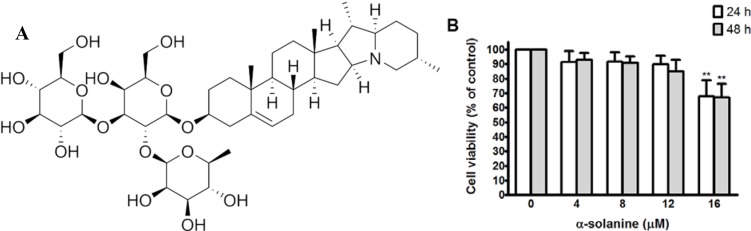
(**A**) Chemical structure of α-solanine. (**B**) Effect of α-solanine on viability of PC-3 cell. Cells were treated with various concentrations of α-solanine for 24 h and 48 h. Cell viability is presented as mean ± S.D. of four independent experiments. ** *p* < 0.01 compared with the untreated control.

The chemical structure of α-solanine is shown in Figure 1A. The cytotoxic effect of α-solanine on human prostate cancer PC-3 cell is illustrated in Figure 1B. As can be seen, treatment with 16 μM α-solanine for 24 h or 48 h significantly decrease the viability of PC-3 cells. Moreover, treatment with solanine at doses of no more than 12 μM for 24 h does not cause cytotoxicity of PC-3 cells. 

### 2.2. Effects of α-Solanine on Inhibiting Migration and Invasion of PC-3 Cells

**Figure 2:**
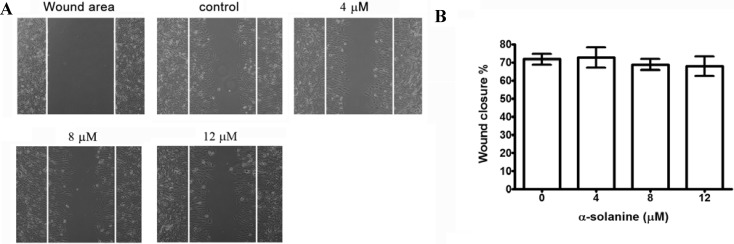
Effect of α-solanine on migration of PC-3 cell. (**A**) Cell monolayers were scraped by a sterile micropipette tip and the cells were treated with various concentrations of α-solanine for 24 h. Cells migrated to the wounded region were photographed (100× magnification). (**B**) The wound area of the cell cultures were quantified in four fields in each treatment, and data were calculated from three independent experiments. Data are presented as mean ± S.D. of three independent experiments.

In view of the toxicity at higher concentration of α-solanine, the inhibitory effect of non-toxic doses of α-solanine on the migration and invasion of PC-3 cells was investigated. After incubation with various concentrations of α-solanine for 24 h, α-solanine shows no suppression on the migration of PC-3 cells to the denuded zone (Figure 2A,B). These results demonstrate that α-solanine does not significantly inhibit the migration of PC-3 cells. 

**Figure 3:**
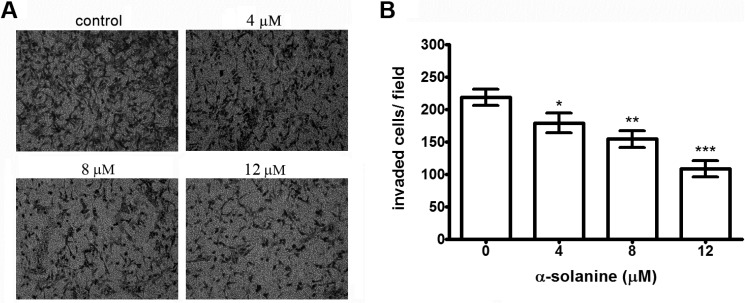
Effect of α-solanine on invasion of PC-3 cell. (**A**) Cells were treated with various concentrations of α-solanine for 24 h and cell invasion assay was performed. The invaded cells were photographed (200× magnification). (**B**) The invaded PC-3 cells were counted in five random fields in each treatment, and data were calculated from three independent experiments. Data are presented as mean ± S.D. of three independent experiments. * *p* < 0.05, ** *p* < 0.01, *** *p* < 0.001 compared with the untreated control.

In order to determine the inhibitory effect of α-solanine on the invasion of PC-3 cells across the extracellular matrix, the cells that invaded through the Matrigel-coated polycarbonate filter in the Boyden chamber were analyzed. The results show that α-solanine suppresses the invasion of PC-3 cells across the Matrigel-coated filter in a dose-dependent manner. Treatment with α-solanine at doses of 4, 8 and 12 μM inhibit 16.9%, 32.1% and 51.4% of cell invasion, respectively (Figure 3A,B), indicating that α-solanine markedly inhibits invasion of PC-3 cells and that such inhibition is not due to any cytotoxicity.

### 2.3. α-Solanine Exerts Reversion Effect on EMT Markers in PC-3 Cells

**Figure 4:**
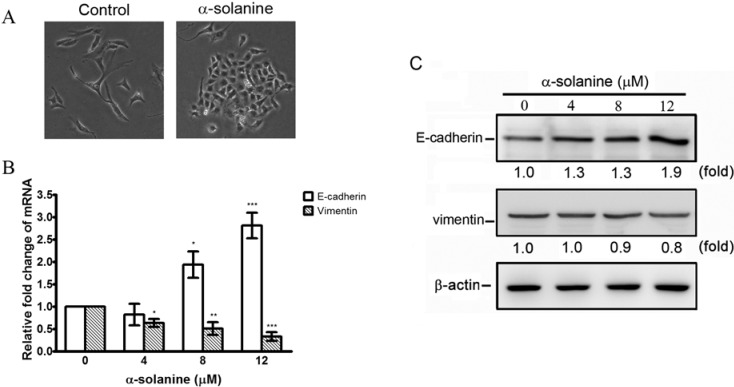
Regulation of epithelial-mesenchymal transition by α-solanine in PC-3 cell. (**A**) Cells were treated with 12 μM of α-solanine for 48 h and cell morphology were photographed (100× magnification). Cells were treated with various concentrations of α-solanine for 24 h and the expressions of E-cadherin and vimentin mRNA (**B**) and protein (**C**) were analyzed by quantitative real-time PCR and Western blotting, respectively. Data are expressed as mean ± S.D. of three independent experiments. * *p* < 0.05, ** *p* < 0.01, *** *p* < 0.001 compared with the untreated control.

EMT is critical for both invasion and metastasis of prostate cancer, so the effect of α-solanine on EMT was explored. Results show that treatment with α-solanine triggers a close cell-cell contact epithelial-like morphology (Figure 4A). α-Solanine significantly induces the expression of E-cadherin and decreases the expression of vimentin in mRNA and protein level in PC-3 cells (Figure 4B,C), suggesting that α-solanine can suppress EMT.

### 2.4. α-Solanine Decreases Expression of MMP-2, MMP-9 and EMMPRIN and Induces Expression of RECK, TIMP-1 and TIMP-2 in PC-3 Cells

**Figure 5:**
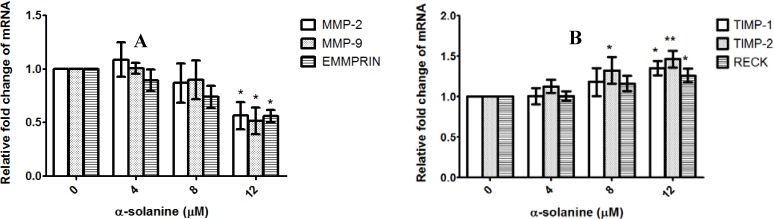
Effect of α-solanine on expressions of MMP-2/-9, EMMPRIN, RECK and TIMP-1/-2 in PC-3 cells. Cells were treated with various concentrations of α-solanine for 24 h and the expressions of MMP-2/-9 and EMMPRIN mRNA (**A**), and RECK and TIMP-1/-2 (**B**) were analyzed by quantitative real-time PCR. Data are expressed as mean ± S.D. of three independent experiments. * *p* < 0.05, ** *p* < 0.01 compared with the untreated control.

Since the expression of MMPs is crucial to ECM degradation, which is required for cell invasion, the effect of α-solanine on the expression of genes involved in ECM degradation was analyzed using quantitative real-time PCR. The primer sequences are listed in Table 1.The results demonstrate that α-solanine suppresses mRNA expression of MMP-2, -9 and EMMPRIN (Figure 5A). In addition, α-solanine elevates the expression of TIMP-1, -2 and RECK which are known to be negative regulators of MMPs (Figure 5B). Taken together, these findings suggest that α-solanine affects the expression of genes involved in proteolytic activation.

**Table 1:** Primer pairs used in Quantitative Real-Time PCR.

Gene	Sequence (5'–3')
MMP-2-F	CTTCCAAGTCTGGAGCGATGT
MMP-2-R	TACCGTCAAAGGGGTATCCAT
MMP-9-F	GGGACGCAGACATCGTCATC
MMP-9-R	TCGTCATCGTCGAAATGGGC
EMMPRIN-F	CTACACATTGAGAACCTGAACAT
EMMPRIN-R	TTCTCGTAGATGAAGATGATGGT
RECK-F	CCTGCATTGCTCGCTGTGTG
RECK-R	CCTGTGGTTTGGGTATGCACCTT
TIMP-1-F	CTTCTGCAATTCCGACCTCGT
TIMP-1-R	CCCTAAGGCTTGGAACCCTTT
TIMP-2-F	AAGCGGTCAGTGAGAAGGAAG
TIMP-2-R	CACACACTACCGAGGAGGG
β-actin-F	CATGTACGTTGCTATCCAGGC
β-actin-R	CTCCTTAATGTCACGCACGAT
miR-21	CGGCGGTAGCTTATCAGACTG A
miR-138	AACGGAGCTGGTGTTGTGAAT C
RNU6B	TTCCTCCGCAAGGATGACACG C

### 2.5. α-Solanine Inhibits Phosphorylation of PI3K, Akt and ERK

**Figure 6:**
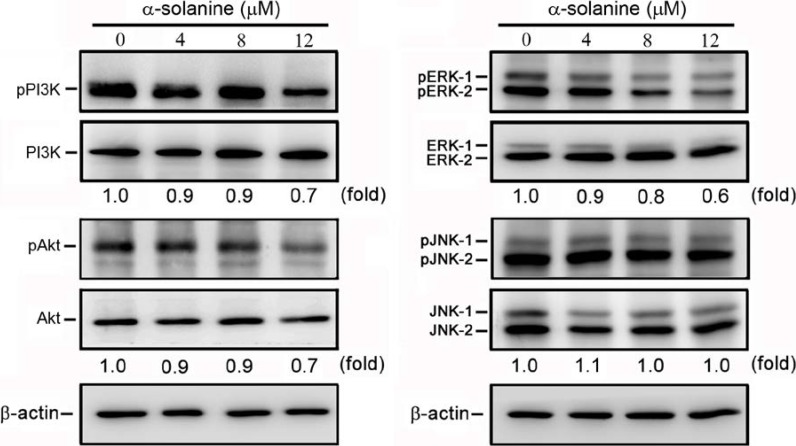
Effects of α-solanine on phosphorylation of PI3K, Akt, ERK1/2 and JNK1/2. Cells were treated with solanine for 24 h and the phosphorylation of PI3K, Akt, ERK1/2 and JNK1/2 were determined by SDS-PAGE and western blotting. β-actin was used as a loading control.

Several studies have indicated that the signaling proteins, including PI3K, Akt and MAPK members are involved in the expression of MMPs and that they induce tumor development and progression [41,45,48,49,50]. In view of these findings, the effect of α-solanine on the phosphorylated status of PI3K, Akt, ERK1/2 and JNK1/2 in PC-3 cells were investigated. The results demonstrate that α-solanine reduces the phosphorylation of PI3K, Akt and ERK1/2 in a dose-dependent manner, but it has no effect on the phosphorylation of JNK1/2 (Figure 6), revealing that the signaling pathways mediated by PI3K/Akt and ERK are suppressed by α-solanine. In addition, previous research has shown that inhibitors of ERK and PI3K/Akt significantly suppressed the expression of MMP-2/9 in PC-3 cells [42], implying that α-solanine-inhibited expression of MMP-2/9 proceeds via the suppression of ERK and PI3K/Akt pathways. Hence, PI3K/Akt and ERK pathways may be the potential targets for suppressing prostate cancer cells metastasis.

**Figure 7:**
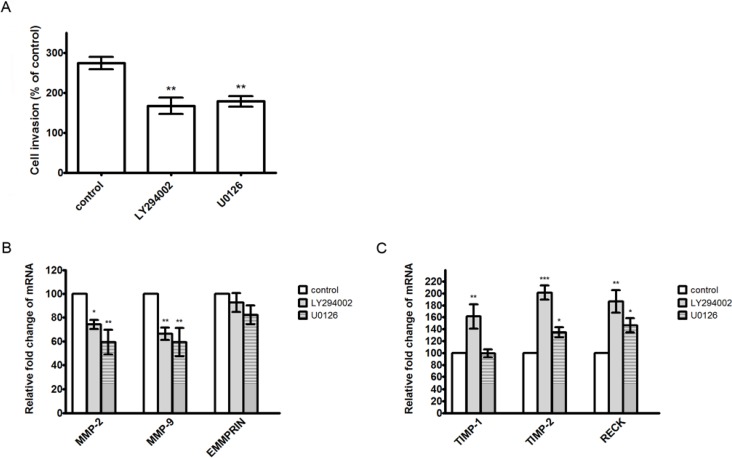
Effects of LY294002 and U0126 on cell invasion and mRNA expression of MMP-2/-9, EMMPRIN, TIMP-1/-2 and RECK. (**A**) Cells were treated with LY294002 (20 μM) or U0126 (40 μM) for 24 h. The cell invasive abilities were performed by Boyden chamber invasion assay. The mRNA expression of MMP-2/-9 and EMMPRIN (**B**) and TIMP-1/-2 and RECK (**C**) were determined by quantitative real-time PCR and expressed as mean ± S.D. of three independent experiments. * *p* < 0.05, ** *p* < 0.01, *** *p* < 0.001, compared with the untreated control.

**Figure 8:**
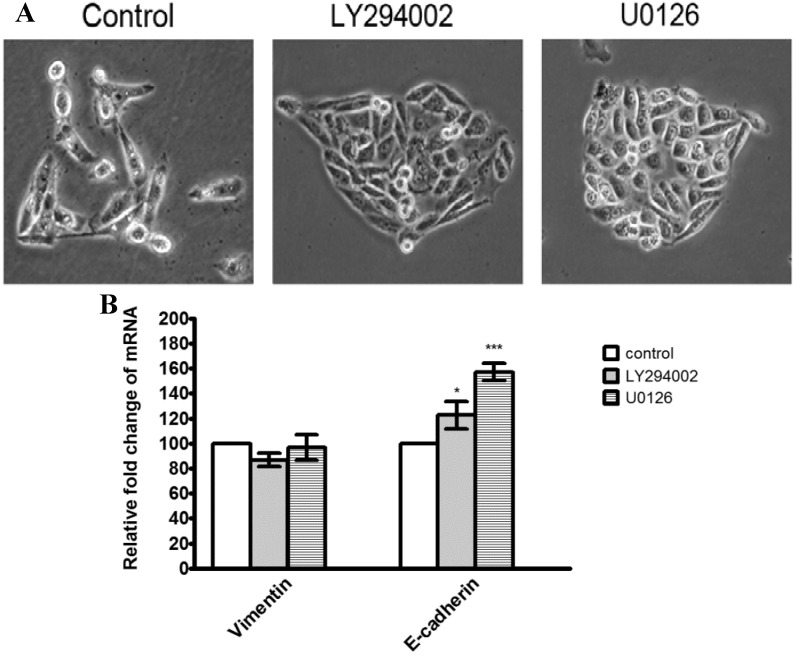
Effects of LY294002 and U0126 on epithelial-mesenchymal transition in PC-3 cell. (**A**) Cells were treated with LY294002 (20 μM) or U0126 (40 μM) for 24 h and cell morphology were photographed (100× magnification). (**B**) The mRNA expression of E-cadherin and vimentin were analyzed by quantitative real-time PCR and expressed as mean ± S.D. of three independent experiments. * *p* < 0.05, *** *p* < 0.001, compared with the untreated control.

To further investigate whether the inhibitory effect of α-solanine on expression of genes involved in proteolytic activation and EMT were through inhibition of the PI3K/Akt and ERK1/2 signaling pathways, PC-3 cells were treated with a PI3K/Akt inhibitor (LY294002; 20 μM) and a ERK inhibitor (U0126; 40 μM) for 24 h. Results show that treatment of LY294002 and U0126 reduced cell invasion and MMP-2/9 mRNA expression, but not affected EMMPRIN expression significantly (Figure 7A,B). Expression of TIMP-2 and RECK were elevated by treatment of LY294002 and U0126, while expression of TIMP-1 was only induced by LY294002 (Figure 7C). In addition, treatment of LY294002 and U0126 induced a close cell-cell contact epithelial-like morphology, and increased expression of E-cadherin, but not affected expression of vimentin (Figure 8A,B). The results reveal that the inhibition of PI3K/Akt and ERK pathways may contribute to the down-regulation of MMP-2/9, and the up-regulation of TIMP-1/2, RECK and E-cadherin expression, and subsequently cause the suppression of cell invasion and the reversal of EMT. 

### 2.6. α-Solanine Down-regulates Expression of miR-21 and Up-regulates Expression of miR-138

**Figure 9:**
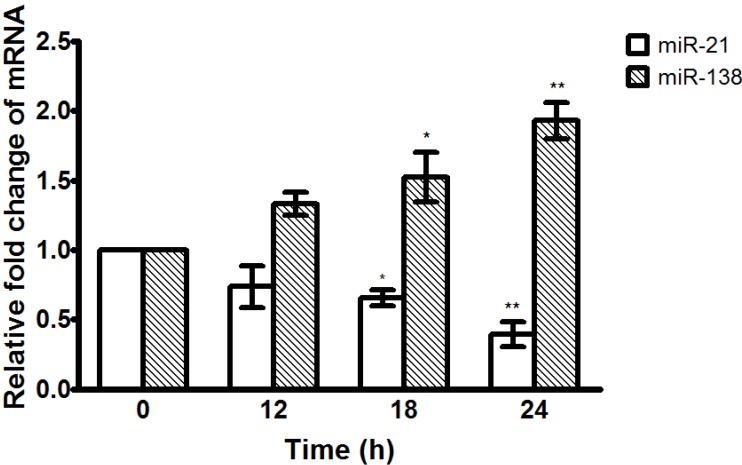
Effects of α-solanine on the expression of miR-21 and miR-138. Cells were treated with 12 μM of α-solanine for 12, 18 and 24 h and the expressions of miR-21 and miR-138 were analyzed by quantitative real-time PCR. Data are expressed as mean ± S.D. of three independent experiments.* *p* < 0.05, ** *p* < 0.01 compared with the untreated control.

To determine the effect of α-solanine on the expression of miR-21 and miR-138, quantitative real-time PCR was performed. The results show that α-solanine down-regulates the expression of miR21 and up-regulates the expression of miR-138 in a dose-dependent manner (Figure 9). The regulation of miR-21 and miR-138 expressions by α-solanine may be involved in the anti-metastatic mechanisms of α-solanine.

### 2.7. Discussion

Nightshade has been commonly used as folk medicine in China to cure sores, carbuncles, swellings, injuries and factures. Recent studies reveal that extract of nightshade possesses anti-tumor activity [1,2,3,4]. Nightshade contains several steroidal alkaloids with anti-tumor effect, including solamargine and α-solanine [6,51]. α-Solanine, one of the major glycoalkaloids in nightshade, has been shown to exhibit toxicity in developing frog embryos, mice livers and in normal human liver cells [6,52,53]. In addition to the toxic effects in normal physiological functions, α-solanine exhibits anti-carcinogenic potentials, such as inhibiting cell growth of various cancer cell lines and inducing apoptosis of human cancer cells [6,7,8]. α-Solanine was also found to inhibit migration and invasion of human melanoma cells, suggesting that α-solanine might possess anti-metastatic potential [10]. This study obtains evidence that α-solanine inhibits the invasion of human prostate cancer PC-3 cells.

The present findings demonstrate that α-solanine suppresses proliferation of PC-3 cells significantly at the concentration of 16 µM. When PC-3 cells are treated with α-solanine at non-toxic doses, cell invasion is inhibited. These results imply that the inhibition of invasion by α-solanine in PC-3 cells is not due to any cytotoxicity.

To elucidate the effect of α-solanine on cell motility, cell migration and invasion are determined by wound healing migration assay and Boyden chamber invasion assay, respectively. Wound healing migration assay has been used very effectively for studying cell migration across a 2D substratum. During migration, cells protrude the leading edge, adhere to the underlying substratum, contraction and detach at distinct regions of the cell. Monolayer wounding assay has also been employed to examine signaling events involved in cell migration, such as Rho GTPases [54,55]. In a Boyden chamber invasion assay, cells adhere to substratum and degrading ECM components, subsequently penetrating and transmigrating ECM. Expression of proteolytic proteases, such as MMPs, is critical for ECM degradation in cell invasion [55]. The present study demonstrates that α-solanine inhibits cell invasion, but has no significant effect on cell migration significantly. These results suggest that the mechanism of cell migration may not be altered by α-solanine. In view of this, whether the inhibitory effect of α-solanine on cell invasion is through reversal of EMT or suppression of MMP expression was investigated.

EMT is critical for cancer metastasis. Induction of EMT can lead cancer cells to invasion of surrounding stroma, intravasation, dissemination and colonization of distant sites. Thus, reversal of EMT is thought to be an effective strategy against cancer metastasis [16]. Here, α-solanine was found to be capable of reversing the process of EMT by decreasing mesenchymal marker vimentin, and increasing epithelial marker E-cadherin. It is also observed that α-solanine treatment induces close cell-cell contact morphology. Taken together, the findings suggest that inhibition of invasion by α-solanine in PC-3 cells may be partly attributed to the reversal of EMT.

During cell invasion, cell protrusion is accomplished by pericellular proteolysis of ECM. Several extracellular proteases, such as MMPs, are participated in proteolytic degradation of ECM and are required for cancer cell invasion. Of these proteases, MMP-2 and MMP-9 play a critical role in the progression of prostate cancer. Expression of MMP-2 and MMP-9 are associated with prostate cancer progression [56,57]. Inhibition of MMP-2 and MMP-9 expression suppresses the metastatic potential of prostate cancer [58]. Besides, other proteins, such as EMMPRIN, are involved in the regulation of proteolytic degradation of ECM in tumor metastasis. EMMPRIN promotes invasion and metastasis processes of prostate cancer cells by inducing the activation of MMPs [23]. RECK acts as an inhibitor of MMPs and can inhibit tumor angiogenesis, invasion and metastasis [24]. The expression of RECK in malignant prostate tumor tissues is lower than that in their normal counterparts and overexpression of RECK down-regulates invasion of prostate cancer cells [59]. Recent studies have demonstrated that the enhancement of RECK expression may suppress cancer cell invasion [59,60]. TIMPs, the regulator of MMPs, are also involved in tumor progression, invasion, metastasis and angiogenesis [61,62]. Increased expression of TIMP-1 has been shown to suppress cell invasion [63,64]. The results of this study suggest that the inhibition of MMP-2/-9 and EMMPRIN expression, and the induction of RECK and TIMP-1/-2 expression may contribute to the anti-invasive effect of α-solanine. 

Many reports have shown that MMP-2 and MMP-9 expression are critically mediated by MAPK members and the PI3K/Akt pathway [45,48,49]. MAPK and PI3K/Akt pathways play an important role in tumor initiation and progression [41,45,50]. This study demonstrates that treatment with α-solanine significantly reduced ERK and PI3K/Akt phosphorylation, suggesting that the signaling pathways mediated by ERK and PI3K/Akt are suppressed by α-solanine. In other words, α-solanine may inhibit the invasion of PC-3 cells by reducing ERK and PI3K/Akt pathways. Moreover, our results suggest that α-solanine inhibits expression of MMP-2/9, vimentin, and induces expression of TIMP-1/2, RECK and E-cadherin can be partly occurred through suppressing ERK or PI3K/Akt pathways. Thus, ERK and PI3K/Akt pathways may be the potential targets for suppressing prostate cancer metastasis. Furthermore, the determination of the therapeutic potential and pharmacodynamic properties of α-solanine in vivo is imperative. The animal study for investigating the effect of α-solanine on prostate cancer metastasis will be carried out in future.

miRNAs have been shown to play a crucial role in tumor biology, including initiation, invasion, metastasis and angiogenesis [30]. miR-21 acts as an oncomir that promotes tumor initiation and invasiveness. It is overexpressed in many types of human cancers, including prostate cancer [31,65]. It has recently been shown that miR-21 regulates invasion of prostate cancer cell by targeting RECK, a key inhibitor of several MMPs [34]. Recent study also indicated that miR-21 regulated EMT by targeting PTEN [35]. The present study demonstrates that α-solanine reverses EMT, elevates RECK expression and down-regulates miR-21 expression, implying that the reversal of EMT and induction of RECK expression by α-solanine may be through the down-regulation of miR-21. miR-138 is regarded as a potential tumor suppressor in various types of cancers [36,37,38]. Several studies have indicated that miR-138 could suppress cancer metastasis by targeting RhoC, ROCK2, MMP-2/-9, FAK, SOX-4 and Hif1a [4,38,66,67]. miR-138 also suppresses EMT by targeting vimentin, ZEB2 and EZH2 [39]. The present results reveal that α-solanine inhibits expression of MMP-2/-9 and vimentin, while up-regulates expression of miR-138, thus suggesting that the inhibitory effect of α-solanine on expression of MMP-2/-9 and vimentin may be through the up-regulation of miR-138.

## 3. Experimental

### 3.1. Reagents and Cell Culture

α-Solanine, dimethyl sulfoxide (DMSO), Tris-HCl, EDTA, SDS, phenylmethylsulfonyl fluoride (PMSF), Nonidet P-40, deoxycholic acid and sodium orthovanadate, were purchased from Sigma-Aldrich (St. Louis, MO, USA). Protein assay kit was obtained from Bio-Rad Labs (Hercules, CA, USA). Powdered Dulbecco’s modified Eagle’s medium (DMEM) was purchased from Gibco/BRL (Gaithersburg, MD, USA). Total RNA extraction kit and PCR kit were from Viogene (Sunnyvale, CA, USA). FastStart Universal Probe Master assay kit was purchased from Roche Applied Science (Indianapolis, IN, USA). Antibodies against E-cadherin, vimentin, Akt and PI3K were purchased from Santa Cruz Biotechnology (Santa Cruz, CA, USA). Antibodies against ERK, JNK and phosphorylated proteins were purchased from Cell Signaling Technology (Danvers, MA, USA). Human prostate cancer cell lines PC-3 was obtained from BCRC (Food Industry Research and Development Institute, Taiwan). Cells were maintained in DMEM supplemented with 10% fetal calf serum, 100 U/mL of penicillin and 100 µg/mL streptomycin, and incubated in a 5% CO_2 _ humidified incubator at 37 °C. For solanine treatment, solanine was dissolved in ethanol and diluted with culture medium (the final concentration of ethanol was less than 0.2%). 

### 3.2. Cell Viability Assay

The assay was performed as described previously [68]. Briefly, cells were seeded in a 96-well plate and treated with α-solanine in triplicate. After 24 and 48 h of incubation, the medium was replaced with fresh medium containing 0.5 mg/mL MTT [3-(4,5-dimethylthiazol-2-yl)-2,5-diphenyltetrazolium bromide]. After 4 h, the supernatants were removed and the resulting MTT formazan was solubilized in DMSO and measured spectrophotometrically at 570 nm.

### 3.3. Wound Healing Migration Assay

The assay was performed as described previously [10]. PC-3 cells were plated in a 12-well plate and grew to confluence. The monolayer culture was then scrape-wounded with a sterile micropipette tip to create a denuded zone (gap) of constant width. After removing the cellular debris with PBS, cells were exposed to various concentrations of solanine for 24 h. PC-3 cells migrated to the wounded region were observed by Olympus CK-2 inverted microscope and photographed (100× magnification). The wound area was measured by the program Image J [69]. The percentage of wound closure was estimated by the following equation: Wound closure % = [1 − (wound area at T_t_/wound area at T_0_) × 100%, where T_t_ is the time after wounding and T_0_ is the time immediately after wounding.

### 3.4. Boyden Chamber Invasion Assay

Boyden chamber invasion assay was carried out as previously [10]. Briefly, the polycarbonate filter (8 µm pore) was pre-coated with Matrigel. After treated with α-solanine for 24 h, cells (1 × 10^4^ cells/well) were added to the upper chamber in serum-free medium. The complete medium (containing 10% FBS) was applied to the lower chamber as chemoattractant. The chamber was incubated for 6 h at 37 °C. At the end of incubation, the cells in the upper surface of the membrane were carefully removed with a cotton swab and cells that invaded to the lower surface of the membrane were fixed with methanol and stained with 5% Giemsa solution. The invaded cells on the lower surface of the membrane filter were scored from five random fields under microscopy (200× magnification).

### 3.5. RNA Extraction and Reverse Transcription PCR and Quantitative Real-time PCR

Total RNA was extraction using total RNA extraction kit according to the manufacturer’s instructions. Total RNA (1 µg) from each sample was subject to reverse transcription with oligo (dT) primers by PCR kit according to manufacturer’s instruction. The mRNA expressions of MMP-2, -9, EMMPRIN, RECK, TIMP-1,-2, E-cadherin and vimentin were determined by quantitative real-time PCR which is conducted in StepOne system (Applied Biosystem, Foster City, CA, USA). Briefly, each amplification mixture (50 μL) contains 10 ng cDNA and 25 μL SYBR Green PCR Master Mix. PCR conditions were as follows: 95 °C for 2 min, 40 cycles at 95 °C for 15 s and 60 °C for 45 s. For detection of miR-21 and miR-138, FastStart Universal Probe Master assay kit (Roche Applied Science) was used according to manufacturer’s instruction, and RNU6B was used as internal control. The primer sequences were listed in Table 1. PCR results were derived using the comparative C_T_ method. 

### 3.6. Western Blotting

After being treated with α-solanine, PC-3 cells were washed twice with PBS and treated with extraction buffer (50 mM Tris-Cl, pH 7.5, 150 mM NaCl, 0.1% SDS, 1% NP-40, and 0.5% deoxycholic acid). The cell extractions were collected and centrifuged at 10,000 g for 10 min at 4 °C, and the supernatants were collected as cell lysates. The cell lysates were subjected to SDS-PAGE, and transferred to nitrocellulose membranes (Millipore, Bedford, MA, USA). The membranes were blocked with 5% (w/v) non-fat milk in PBS containing 0.1% Tween-20, and then blotted with primary antibody. Subsequently, the membranes were incubated with an appropriate secondary antibody (horseradish peroxidase-conjugated goat anti-mouse or anti-rabbit IgG). The immuno-detected proteins were then revealed by enhanced chemiluminescence.

### 3.7. Statistical Analysis

Data were expressed as mean ± standard deviation. Statistical significance was analyzed by one-way ANOVA. If the significance was observed, the Dunnett’s *post-hoc* test was used to determine the difference between treatment groups and untreated group, with values of *p* < 0.05 considered statistically significant.

## 4. Conclusions

In conclusion, this study demonstrates the inhibitory effect of α-solanine on the invasion of human prostate cancer PC-3 cells in a dose-dependent manner, which is also accompanied by the reversal of EMT. The regulation in the expression of MMP-2/9, vimentin and RECK by α-solanine is possibly caused by the expression of miR-138 and miR-21. These effects may contribute to the inhibition of invasion in PC-3 cells by α-solanine. The present findings reveal a novel therapeutic potential of α-solanine for suppressing invasion of prostate cancer cell. 
